# Performance Characteristics of Oncomine Focus Assay for Theranostic Analysis of Solid Tumors, A (21-Months) Real-Life Study

**DOI:** 10.3390/diagnostics13050937

**Published:** 2023-03-01

**Authors:** Jessica Bamba-Funck, Emmanuelle E. Fabre, Marianne Kambouchner, Olivier Schischmanoff

**Affiliations:** 1 Laboratory of Biochemistry, Hôpital Avicenne, Hôpitaux Universitaires Paris-Seine-Saint-Denis, Assistance Publique—Hôpitaux de Paris, F-93000 Bobigny, France; 2 Laboratory for Vascular Translational Science, LVTS, UMR INSERM 1148, UFR SMBH, Université Sorbonne Paris Nord, F-93000 Bobigny, France; 3 Signaling, Microenvironment and B Cell Malignancies, SIMEL, UMR INSERM U978, UFR SMBH, Université Sorbonne Paris Nord, 8, F-93000 Bobigny, France; 4 Department of Pathology, Hôpital Avicenne, Hôpitaux Universitaires Paris-Seine-Saint-Denis, Assistance Publique—Hôpitaux de Paris, F-93000 Bobigny, France

**Keywords:** next generation sequencing (NGS), solid tumors, single nucleotide variation (SNV), copy number variation (CNV), fusion transcript, long-term follow-up

## Abstract

Next generation sequencing analysis is crucial for therapeutic decision in various solid tumor contexts. The sequencing method must remain accurate and robust throughout the instrument lifespan allowing the biological validation of patients’ results. This study aims to evaluate the long-term sequencing performances of the Oncomine Focus assay kit allowing theranostic DNA and RNA variants detection on the Ion S5XL instrument. We evaluated the sequencing performances of 73 consecutive chips over a 21-month period and detailed the sequencing data obtained from both quality controls and clinical samples. The metrics describing sequencing quality remained stable throughout the study. We showed that an average of 11 × 10^6^ (±0.3 × 10^6^) reads were obtained using a 520 chip leading to an average of 6.0 × 10^5^ (±2.6 × 10^5^) mapped reads per sample. Of 400 consecutive samples, 95.8 ± 16% of amplicons reached the depth threshold of 500X. Slight modifications of the bioinformatics workflow improved DNA analytical sensitivity and allowed the systematic detection of expected SNV, indel, CNV, and RNA alterations in quality controls samples. The low inter-run variability of DNA and RNA—even at low variant allelic fraction, amplification factor, or reads counts—indicated that our method was adapted to clinical practice. The analysis of 429 clinical DNA samples indicated that the modified bioinformatics workflow allowed detection of 353 DNA variants and 88 gene amplifications. RNA analysis of 55 clinical samples revealed 7 alterations. This is the first study showing the long-term robustness of the Oncomine Focus assay in clinical routine practice.

## 1. Introduction

Theranostic analysis of solid tumors gains continuous complexity requiring the search for point mutations, short insertions or deletions, copy number variations, or gene fusions in always larger panels of genes [1,2]. Next generation sequencing (NGS) allows to keep up with the search for a growing number of genetic alterations in limited amounts of biologic material with a temporality compatible with clinical needs. NGS may allow reporting results to clinicians in a minimum of five days from the acquiring of formalin-fixed, paraffin-embedded (FFPE) samples. Implementation of a new NGS method follows well-defined procedures that are usually defined by academic societies or by state medical agencies [3,4]. Following the new NGS method set up, identification of factors that contribute to alter data quality over time is of critical importance. Indeed, several environmental, material, or human factors may impact NGS long-term performances (i.e., analytical sensitivity), possibly affecting the choice of clinical treatments [2,5]. Indeed, the initial validation does not ensure that the method will be robust enough to produce accurate results throughout the instrument lifespan.

Two major NGS methods are commonly used in diagnostic laboratories. Although both methods are based on sequencing by synthesis, Thermo Fisher and Illumina platforms use different principles for sequencing. Thermo Fisher Ion Torrent^TM^ technology directly converts nucleotide sequences into digital information using a semiconductor chip measuring variation of pH [6]. Illumina “bridge amplification” technology is based on incorporation and detection of fluorescent nucleotides in DNA fragments immobilized on a glass slide [7]. A recent study reported that both technologies achieved comparable NGS performances (i.e., mean read coverage, mean coverage uniformity, and variant allele frequency) and are suitable for diagnosis purpose [8].

Our laboratory implemented the Oncomine^TM^ Focus Assay (OFA) on an Ion S5XL (ThermoFisher, Les Ulis, France). This assay is dedicated to the identification of all types of theranostic variations in FFPE tissues and enables the concurrent analysis of DNA and RNA to detect variations in 52 genes in solid tumors [9,10]. To our knowledge, no study has been dedicated to investigate the evolution of the performances of OFA over a long period of time. 

The aim of this study was to evaluate retrospectively the evolution of the performances of the OFA during a 21-month period in order to verify whether it is suitable for hospital routine practice. 

## 2. Materials and Methods

### 2.1. Sample Section

This study was performed at Hospital Avicenne, Molecular and Biochemistry Laboratory (93000-Bobigny-France), from January 2020 to September 2021 and approved by the Local Ethic Comity (Avicenne Hospital). Formalin-fixed, paraffin-embedded (FFPE) tissues originating from surgical biopsies were systematically reviewed by a skilled pathologist who determined the tumor cellularity. Commercial quality samples were used for DNA assay validation (Quantitative Multiplex Reference Standard (cat. no. HD200, Horizon Diagnostics, Waterbeach, Cambridge, UK), *EGFR* Gene-Specific Multiplex Reference Standard (FFPE) 5% Variant Allelic Fraction (VAF) (cat. no. HD300, Horizon Diagnostics), Structural Multiplex Reference Standard (FFPE) (cat. no. HD789, Horizon Diagnostics). RNA assay characteristics performances were assessed using *ALK-RET-ROS1* Fusion FFPE RNA (cat. no. HD784, Horizon Diagnostics) and Seraseq FFPE Tumor Fusion RNA Reference Material v4 (cat. no. SER0710-0496, Seracare, Ozyme, Saint Cyr l’Ecole, France).

Anonymized FFPE patient samples or quality control (QC) samples originating from 73 consecutive sequencing runs were used for quality metrics assessment. DNA alterations were analyzed from 420 patient samples originating from various tumors including 277 lung, 64 colon, 34 skin, and 45 other tissues (18 pancreas, 16 bile ducts, 5 breast, 3 thyroids, and 3 spleens) over a 21-month period. RNA fusions and exon skipping were analyzed from 55 tissue samples (45 lung, 8 bile duct, 1 pancreas, and 1 thyroid) over a 12-month period.

### 2.2. DNA and RNA Extraction

DNA and RNA were isolated using the Maxwell 16 Instrument using Maxwell^®^ RSC DNA FFPE Kit and Maxwell^®^ RSC RNA FFPE Kit, respectively, according to the manufacturer’s protocols (Promega, Madison, WI, USA). DNA and RNA quantification were performed on a Qubit 2.0 Fluorometer using Qubit dsDNA and Qubit HS RNA assay, respectively (Thermo Fisher, Les Ulis, France).

### 2.3. Library Preparation and Sequencing

For RNA assay, prior to library preparation, cDNA were synthetized using SuperScript VILO cDNA synthesis kit (catalog no. 11756050, Thermo Fisher). Library preparations were carried out using Oncomine Focus Assay, Chef-Ready Library (Thermo Fisher) using an Ion Chef Instrument (Thermo Fisher) following the manufacturer’s instructions using a total of 10ng of DNA or 2ng of RNA.

The DNA panel is designed to identify hotspot mutations of 35 genes (*AKT1*, *ALK*, *AR*, *BRAF*, *CDK4*, *CTNNB1*, *DDR2*, *EGFR*, *ERBB2*, *ERBB3*, *ERBB4*, *ESR1*, *FGFR2 FGFR3*, *GNA11*, *GNAQ*, *HRAS*, *IDH1*, *IDH2*, *JAK1*, *JAK2*, *JAK3*, *KIT*, *KRAS*, *MAP2K1*, *MET*, *MTOR*, *NRAS*, *PDGFRA*, *PIK3CA*, *RAF1*, *RET*, *ROS*, and *SMO*), copy number variations of 19 genes (*ALK*, *AR*, *BRAF*, *CCND1*, *CDK4*, *CDK6*, *EGFR*, *ERBB2*, *FGFR1*, *FGFR2*, *FGFR3*, *FGFR4*, *KIT*, *KRAS*, *MET*, *MYC*, *MYCN*, *PDGFRA*, and *PIK3CA*), fusions drivers of 21 genes (*ABL1*, *ALK*, *AKT1*, *AXL*, *BRAF*, *ERBB2*, *ERG*, *ETV1*, *ETV4*, *ETV5*, *FGFR1*, *FGFR2*, *FGFR3*, *NTRK1*, *NTRK2*, *NTRK3*, *PDGFRA*, *PPARG*, *RAF1*, *RET*, and *ROS1*), and exon skipping of 2 genes (*EGFR* and *MET*). 

RNA and DNA libraries were equalized at 100pM using the Ion Chef instrument and pooled before templating (Ion 510™ & Ion 520™ & Ion 530™ Kit—Chef, cat. no. A34461, Thermo Fisher). Eight DNA samples or both eight DNA samples and eight RNA samples were loaded on a 520 chip (Ion 520™ Chip Kit, catalog no. A27762 Thermo Fisher). Sequencing was performed on an Ion S5XL instrument (Thermo Fisher).

The Tumor Hot Spot assay (THS, Multiplicom, Les Ulis, France), on a MiSeq sequencer (Illumina, Paris, France), was used for OFA inter-run variability comparison.

### 2.4. Bioinformatic Pipeline

For each run, quality metrics were assessed including chip loading density, number of total reads, percentage of clonality, percentage of adapter dimer, percentage of low quality, read length, and alignment of the reads to the hg19 human reference genome (Torrent server, version 5.12, Thermo Fisher). The “coverage Analysis” plugging was applied to assess the quality of sample sequencing. Each sample must reach strict validation criteria, i.e., yield a minimum of 400,000 reads, 98% of the amplicons with a minimal sequencing depth of 500X, 90% of the reads located within the target region boundaries, 80% of the amplicons being read from end-to-end, and 90% of the amplicons being read without strand bias. 

For DNA variant annotation, the Ion Reporter software was carried out (version 5.10). The following default parameters were modified from the “Oncomine Focus w2.4—DNA—Single Sample” workflow: variants were reported with allele view, complex variants were allowed, down-sampled to coverage was set to 5000 reads, and the variants were generated at a minimum VAF of 0.03. The hotspot file was also modified: some hotspot mutations were added to report minor variants within *BRAF*, *KRAS*, and *NRAS* genes (Appendix A). The minimal value for VAF detection was decreased to 0.02 for positions of theranostic interest within *BRAF*, *EGFR*, *KRAS*, and *NRAS* genes. CNV were reported by the pipeline if the MAPD (median absolute pairwise difference) was below 0.4 and if the amplification factor was above 4. 

For RNA variant annotation, the “Oncomine Focus—520—w2.4—Fusions—Single Sample” workflow was used with default parameters. The validation criteria for RNA samples were as follows: each sample must generate at least 20,000 reads and have a minimum mean read length of 50 pb. Additionally, at least three of the five RNA internal controls (TBP, LRP1, ITGB7, MYC, and HMBS) must be called. Finally, RNA alterations were reported only if a minimum number of reads was reached: 20 for targeted fusions, 250 for non-targeted fusions, and 120 for exon skipping. 

For the inter-run variability comparison study, BAM files generated by the MiSeq instrument were analyzed using a tailored Sophia Genetics pipeline (Sophia Genetics, Lausanne, Switzerland). Variant calls were confirmed using the Integrative Genomics Viewer tool (Broad Institute, Cambridge, MA, USA) when necessary.

### 2.5. Immunohistochemistry

Immunohistochemistry for *ROS1*, *ALK*, and *ERBB2* was performed as previously described [11,12]. Tissues were incubated with rabbit anti-*ALK* monoclonal antibody prediluted at 1:2 (clone K52076; Dako, Agilent, les Ulis, France) and anti-*ROS1* monoclonal antibody (clone 1A4; Origene, Clinisciences, Nanterre, France). *ERBB2* status was assessed using the HercepTest™ (kit K5207, Dako-Agilent, Les Ulis, France).

## 3. Results

### 3.1. Sequencing Performances

**Figure 1:**
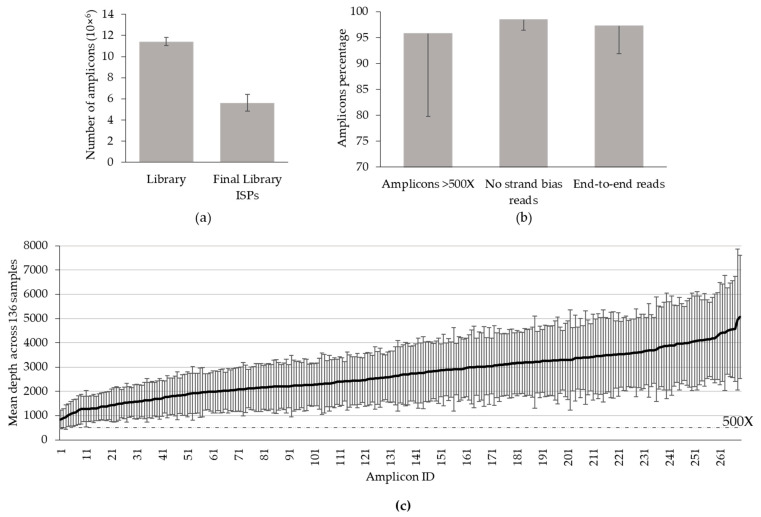
Quality metrics of the OFA DNA panel. (**a**) Number of amplicons per 520 chip for DNA sequencing. Library: live wells with library template sequence; Final library ISPs (Ion Sphere™ Particle): sequence available for analysis after filtering (following removal of polyclonal ISPs, low quality sequences, and adapter dimers) (*n* = 73 chips). (**b**) Percentage of amplicons. Amplicon >500X: percentage of amplicons with at least 500X reads; No strand bias reads: percentage of amplicons that did not show a bias. An individual amplicon has read bias if the percentage of forward or reverse reads to total reads is greater than 70%; End-to-end reads: the percentage of on target reads that fully covered their assigned sequence from end-to-end (*n* = 400 samples). (**c**) Mean amplicon coverage across patient samples (*n* = 136 samples). The amplicon IDs are listed in Appendix B. Errors bars represent the standard deviation.

The quality metrics of the OFA DNA panel were assessed using 73 consecutive runs on 520 chips including QC and clinical samples. An average of 11 × 10^6^ (±0.3 × 10^6^; CV = 3.4%) reads were initially generated per run. The polyclonal ion sphere particles (ISP), primer dimers, low quality reads, and test quality sequences were then filtered out (5.8 × 10^6^ ± 0.8 × 10^6^ reads). Hence, 49 ± 7% (CV = 13%) of the amplicons presented the required quality for further bioinformatics analysis (Figure 1a). Among an average number of sequenced bases of 77 × 10^6^ (±2.5 × 10^6^), 72 × 10^6^ (±2.4 × 10^6^) bases were sequenced with a quality score of 20 (Q20). The mean sequencing depth for a given on-target base was 2246X ± 939X. All the metrics, independent of DNA quality, were measured at each run during the 21-month period and did not reveal any loss of performance over time, as assessed by the low coefficients of variation. 

For each sample, an average of 6.0 × 10^5^ (±2.6 × 10^5^; CV = 44%) reads were mapped to *hg19* genome reference and 98.2 ± 7.1% of the reads were aligned over a target region. The mean read length was 114 bp (± 4 bp). The uniformity of base coverage, defined as the percentage of on-target bases covered by at least 20% of mean coverage depth, was 98.5 ± 8.4%. 

Analysis of 400 consecutive samples revealed an average of 95.8 ± 16% (CV = 16.7%) of amplicons with at least 500X, 98.4 ± 2.0% (CV = 2.0%) of amplicons with no strand bias, and 97.3 ± 5.4% (CV = 5.6%) of amplicons with end-to-end sequencing (Figure 1b). A total of 64 of these 400 samples (16%) failed to reach Thermo Fisher quality requirements due to insufficient depth coverage (*n* = 58), strand bias (*n* = 2), or default of end-to-end sequencing (*n* = 4) (Figure 1b). 

A more detailed analysis of 136 consecutive samples indicated that 97.8% (133/136) of all samples reached an average depth of 500X with a mean sequencing depth of 2695X ± 432X across all amplicons. Some amplicons reached 5066X ± 2535X (OCP1_MET_8). The amplicons more likely to be covered with less than 500X were OCP1_DCUN1D1_9: 845X ± 386X, OCP1_KRAS_10: 892X ± 403X, and OCP1_AR_8: 938X ± 506X (Figure 1c).

### 3.2. Long Term Inter-Run Variability for SNVs and Indels

**Figure 2:**
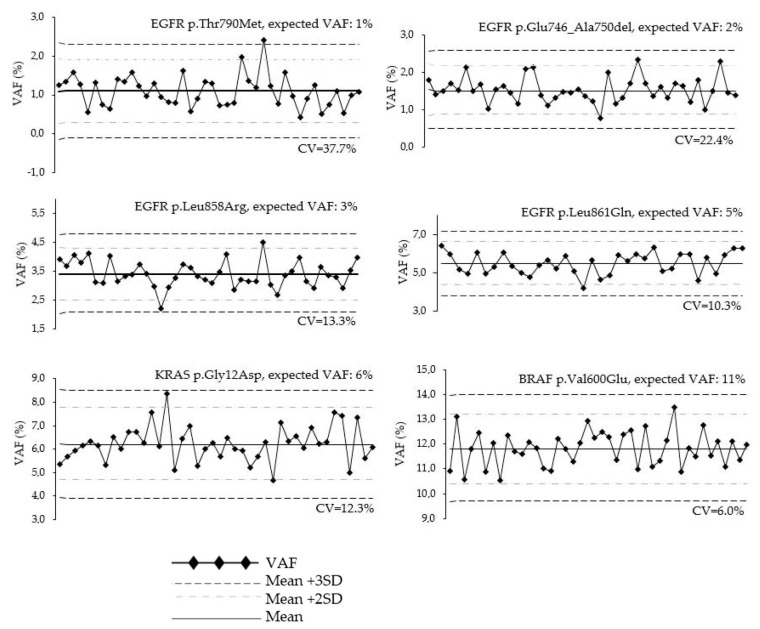
LeveyJennings plots of VAF obtained for six representative variants from HD200 and HD300 QC materials on S5XL instrument over a 21-month follow-up period.

Inter-run variability for SNVs and indels analysis was performed using both QC materials and patient samples. To assess the inter-run performance of the OFA using S5XL instrument, different QC materials were analyzed (HD300: *n* = 42 runs; HD200: *n* = 35 runs). All expected mutations in the reference material were called at expected VAFs. Coefficient of variation of measured VAF ranged from 5.6% (p.His1047Arg, expected VAF = 17.5%) to 37.7% (p.Thr790Met, expected VAF = 1%) (Table 1; Figure 2 and Figure S1). The follow-up of the VAF QC material by Levey–Jennings charts assessed the low inter-run variability for VAF ranging from 1% to 11%. Furthermore, no drift of VAF values was observed. Indeed, during the 21-month period, for the lowest expected VAF (*EGFR* p.Thr790Met), only one value (2.3%) overpassed the limit of 3SD (Figure 2).

These data were also compared to a similar inter-run variability study performed using the THS on the Miseq instrument (HD300: *n* = 42 runs; HD200: *n* = 43 runs). The comparison of the VAF obtained for expected variants indicated a strong correlation between both methods (Pearson’s r = 0.990) (Table 1).

**Figure 3:**
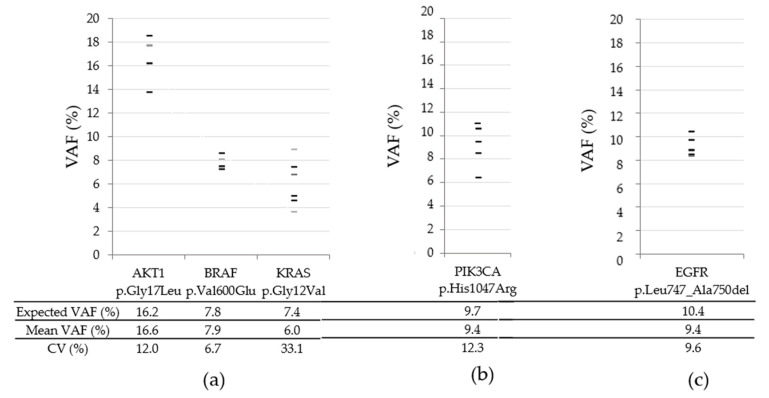
Inter-run variability of variant quantification in clinical samples. (**a**) colorectal tissue; (**b**,**c**) lung tissues. Expected VAF were determined using THS assay (*n* = 1); mean VAF were determined using OFA (*n* = 6).

To further evaluate inter-run variability, clinical samples from colorectal and lung tissues were analyzed within six different runs. Indeed, *PIK3CA* p.His1047Arg, *EGFR* p.Leu747_Ala750del, *AKT1* p.Gly17Leu, *BRAF* p.Val600Glu, and *KRAS* p.Gly12Val mutations previously detected using the THS assay were called at each run using the OFA (Figure 3). The VAF coefficients of variation ranged from 6.7% (*BRAF* p.Val600Glu) to 33.1% (*KRAS* p.Glu12Val).

### 3.3. Detection of SNVs and Indels

**Figure 4:**
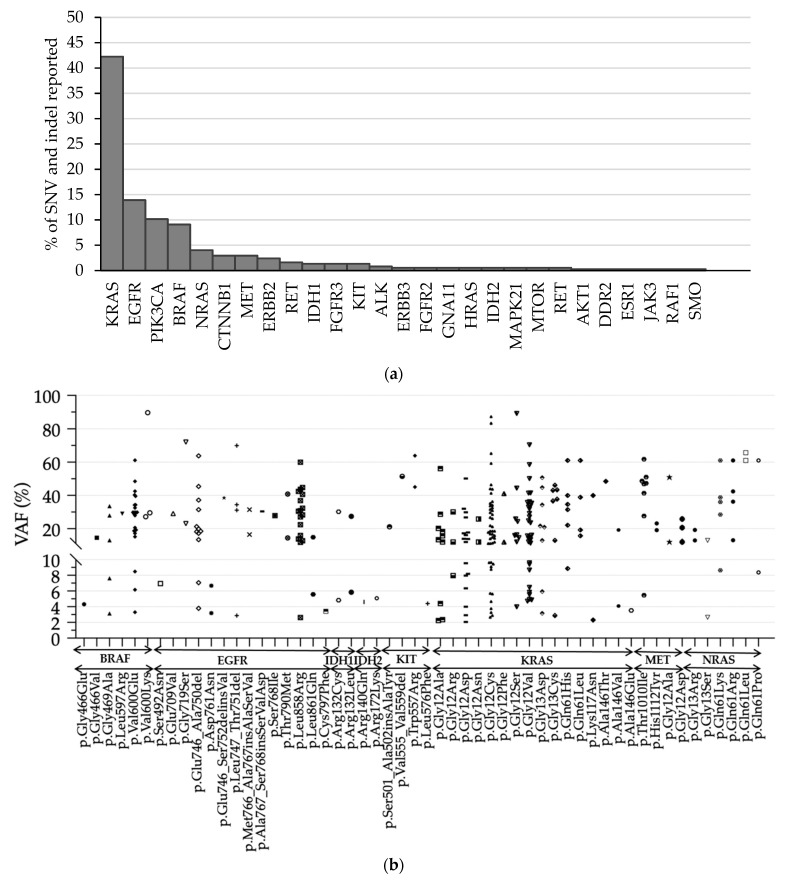
Reported mutations in clinical samples during 21-month follow-up. (**a**) Distribution of all mutations detected across DNA clinical samples (276 lung, 64 colon, and 34 skin tissue samples); (**b**) VAF of reported mutations in *BRAF*, *EGFR*, *IDH1*, *IDH2*, *KIT KRAS*, *MET*, and *NRAS* genes. For a given mutation, a unique symbol was used to indicate the VAF values obtained for different clinical samples.

During the 21-month study of the OFA, the pipeline allowed to detect a large variety of mutations at a broad range of VAF. Among 420 consecutive DNA clinical samples, the pipeline reported SNV or indel in 294 samples (70%). The distribution of the most frequently mutated genes was *KRAS* 42.2% (*n* = 158), *EGFR* 13.9% (*n* = 52), *PIK3CA* 10.2% (*n* = 38), *BRAF* 9.1% (*n* = 34), *NRAS* 4.0% (*n* = 15), and *CTNNB1* 2.9% (*n* = 11) (Figure 4).

During our study, 353 DNA variants were detected. Sixty-four mutations had a VAF comprised between 2 and 10%. The original pipeline was designed to detect hotspot mutations with a VAF above 3%. The modification of the pipeline allowed the detection of 10 (3.4%) mutations with VAF below 3% with potential theranostic impact: *EGFR* p.Leu747_Thr751del (2.9%), p.Leu858Arg (2.6%); *KRAS* p.Gly12Ala (2.4% and 2.4%) p.Gly12Asp (2.1% and 2.9%), p.Gly12Cys (2.7%), p.Gly13Cys (2.9%), p.Lys117Asn (2.3%); and *NRAS* (2.7%).

### 3.4. Performances of CNV Detection

To assess inter-run variability, 20 QC samples were analyzed in different runs. The MAPD scores ranged from 0.272 to 0.356, assessing a low read coverage noise (Table 2). Furthermore, the inter-run variability of MAPD was low (mean: 0.305 ± 0.032), indicating the long-term performance stability of CNV determination. *MET* and *MYCN* gene amplifications were systematically reported close to expected levels. In addition, the variation of inter-run CNV quantification remained low as assessed by the low coefficient of variation for each CNV level. No drift of the measured CNV value was observed, indicating that the performance of the method remained stable over time. 

This study was extended to three clinical samples, which were sequenced twice. *KRAS*, *EGFR*, *FGFR2*, *CCND1*, *CDK4*, and *ERBB2* genes amplifications were reported in the two runs with equivalent amplification rates.

**Figure 5:**
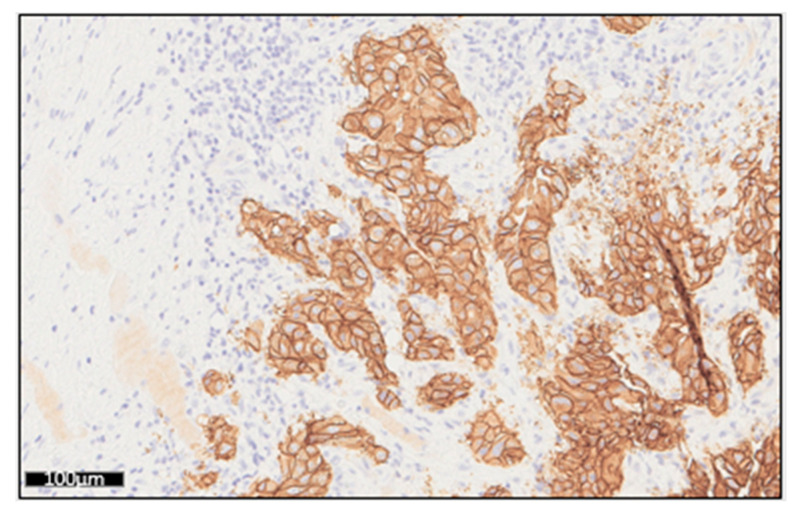
Overexpression of ERRB2 in a case of gene amplification. Immunocytochemistry analysis of ERRB2 protein expression in a lung adenocarcinoma (Patient 1).

Furthermore, we confirmed CNV detection of *ERBB2* gene by studying protein expression level using immunochemistry assay in four lung tumors samples (Table 3; Figure 5). Nuclei were stained in blue (hematoxylin), cytoplasm of positives cells for ERBB2 were stained in brown, while negative cells remained unstained. A large view of bone tissue section showing negative staining for ERBB2 is shown in Figure S2.

### 3.5. Detection of Gene Amplification in Routine Practice

**Figure 6:**
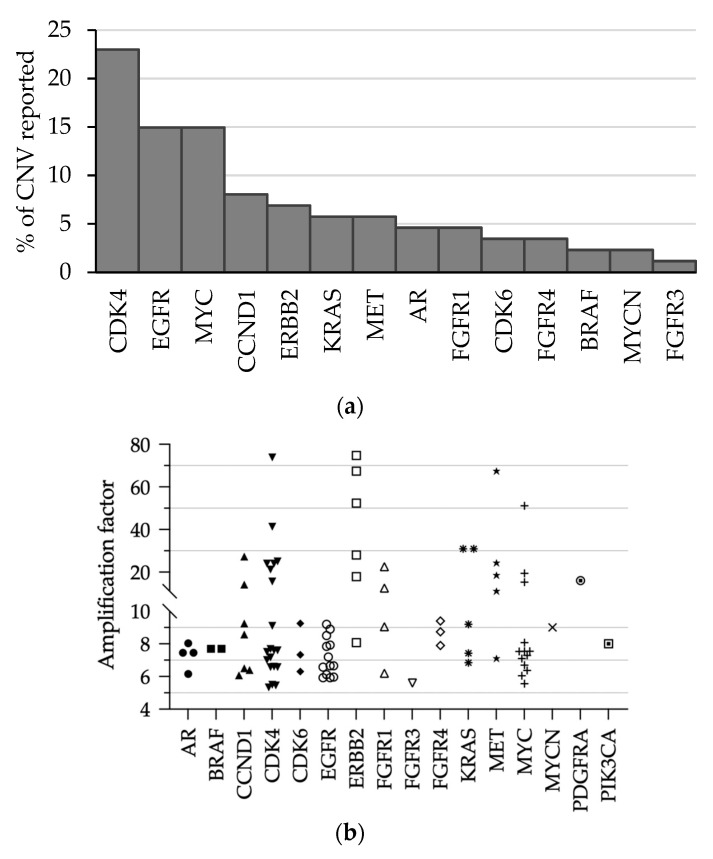
Gene amplification in clinical samples during the 21-month follow-up. (**a**) Gene distribution of CNV; (**b**) CNV detected in 420 clinical samples. For a given gene, a unique symbol was used to indicate the amplification factors obtained for different clinical samples.

The pipeline revealed 88 (20.7%) gene amplifications among 420 clinical samples. These amplifications were detected in 56 lung, 6 colon, 3 bile duct, 3 pancreas, 2 skin, and 2 breast samples. The genes exhibiting most frequently CNV were *CDK4* (*n* = 20), *EGFR* and *MYC* (*n* = 13 each), *CCND1* (*n* = 7), *ERBB2* (*n* = 6), and *KRAS* and *MET* (*n* = 5 each) (Figure 6a). The amplification factors reported by the OFA pipeline ranged from 5 to 75. Amplification factors remained below 6x in eight cases and were not reported to clinicians (7%) (Figure 6b).

### 3.6. Gene-Fusion and Exon-Skipping Detection

RNA samples from HD784 and Seracare QC materials were sequenced in 29 and 7 runs, respectively. Quality criteria were satisfactory for each sample, allowing further data analysis. Expected RNA fusions and exon skipping variations were systematically detected. Fusion reads represented 1.6 to 17.8% of total reads counts (Table 4). The inter-run CV of the percentage of total reads counts ranged from 7.9 to 38.4%. No drift in the detection of both fusions and exon skipping was observed, indicating that the performance of the method remained stable over time.

**Figure 7:**
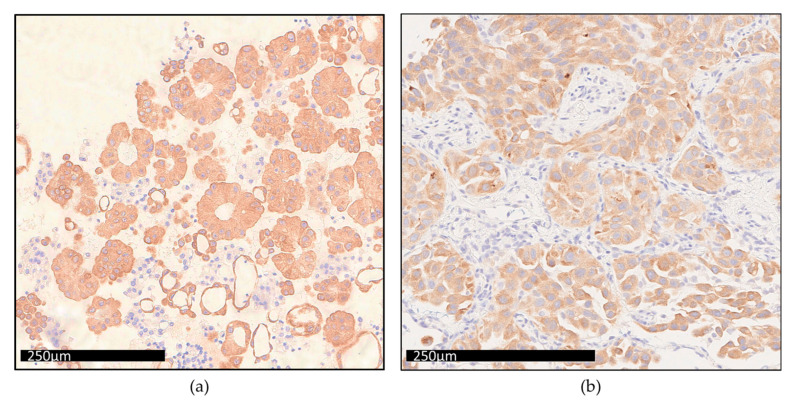
Validation of ALK and ROS1 gene rearrangements by immunohistochemistry analysis. (**a**) Tumor cells strong and diffuse cytoplasmic expression for ALK in bronchoalveolar lavage obtained from papillary adenocarcinoma. (**b**) Tumor cells strong positive staining for ROS1 in a tissue section from lung adenocarcinoma.

To complete the validation of *MET* exon 14 skipping detection, we analyzed RNA material from two patients with *MET* alterations previously detected at DNA level with the THS assay (*MET* c.3082 +1G>C and *MET* c.2888-2A>C). These mutations were detected at the RNA level with the OFA in two independent experimentsIn order to validate *ALK* and *ROS* detection, two gene fusions determined by OFA [*(EML4*(6)—*ALK*(20) (reads counts: 2024, total mapped fusion reads: 49,617) and *CD74*(6)—*ROS1*(34) (reads counts: 1027, total mapped fusion reads: 31,980)] were confirmed by immunochemistry (Figure 7). Nuclei were stained in blue (hematoxylin), cytoplasm of positives cells for ALK and ROS1 were stained in brown, while negative cells remained unstained. Negative ROS1 staining of non-tumoral lung tissue is shown in Figure S3.

### 3.7. Detection of Fusion and Exon Skipping in Routine Practice

Among 55 RNA clinical samples, 11 samples were not suitable for analysis because of poor sequencing quality and no RNA modification was detected for 37 samples. The OFA pipeline detected three *MET* exon skipping alterations, two *ROS* fusion transcripts in lung tissues (*CD74*(6)—*ROS1*(34) and *SLC34A2*(13)—*ROS1*(34)), one *ALK* fusion transcript in lung tissue (*EML4*(6)—*ALK*(20)), and one *PAX8* fusion transcript in thyroid tissue (*PAX8*(9)—*PPARG*(2)).

## 4. Discussion

Theranostic analysis of tumors becomes continuously more complex requiring the study of ever more larger panels of genes in search of point mutations, short insertions and deletions, CNV, or gene fusions. In addition, the development of less invasive sampling techniques such as needle biopsy lead to the decrease of available material. Despite these limitations, genetic analysis must remain compatible with a rapid determination of therapeutic strategy. NGS is the method of choice to fulfill these challenges, assuming that optimal analytical performances are maintained over time.

The aim of this study was to determine whether the analytical performances of the Oncomine Focus Assay remain suitable for clinical practice over a 21-month period. For this purpose, we evaluated the sequencing results obtained from both QC and clinical samples along 73 consecutive runs on an Ion S5 XL sequencer. During this period, we observed no technical failure and the inter-runs variability of critical parameters was adapted to clinical practice.

We first observed that the template generation was reproducible over time. Indeed, the coefficient of variation of the chips loading was 3.4% (mean: 11 × 10^6^ amplicons). Subsequent bioinformatic processing indicated that 49% (CV = 13%) of these amplicons had a sufficient quality for further processing. Additionally, we showed that 6.0 × 10^5^ (CV = 44%) reads per sample were mapped to hg19 reference genome. These CV reflected also the variation in DNA quality of samples. However, our results were consistent with other studies using the same technique. Indeed, Bartlett et al. showed that intra-laboratory variability of mapped reads generation may range from 0.18 × 10^6^ to 1.6 × 10^6^ (10). In our study, the quality of mapped reads within both QC and clinical samples was maintained over time, assessed by the low variability of percentage of end-to-end reads (97.3%; CV = 5.6%) and reads without strand bias (98.4%; CV 2.0%).

The main point of variation, influenced by DNA quantity, was the percentage of amplicons reaching 500X per sample [2]. In our routine practice, although a depth below 500X may remain informative when mutations are at a high allelic frequency, we consider that the depth should reach 500X to validate a negative result. We showed that, for a given sample, 95.8% (CV = 16.7%) of the amplicons reached a depth of 500X. Moreover, all amplicons were individually covered with at least 845X (Figure 1). These results are comparable with those of Williams et al.’s study reporting 89.7% of samples with an average amplicon coverage above 500X [9]. In our analysis, all hotspot regions were sequenced with at least 1574X. Of note, the amplicons sequenced with a lower depth targeted only CNV positions (Figure 1c; Appendix B). We successfully sequenced 98% of clinical samples, a proportion comparable to the 95% described by Williams et al. [9]. These data suggest (i) the tissular heterogeneity of samples did not affect the quality of the sequencing and (ii) the performance of sequencing did not decline over time. Considering that numerous samples were tissue biopsies, we show that the OFA is adapted to this limited amount of material.

The call of the mutations with the Ion Reporter software could fail because of inadequacy of the bioinformatic pipeline. Therefore, we modify some parameters of the workflow. Clinical purpose led us to use samples with a very low acid nucleic concentration, potentially causing limited sequencing depth. In order to improve robustness of our analysis, we modified the hotspot file (Appendix A). We set the detection threshold down to 3% of AF for SNV and indel, and down to 2% for driver variations in BRAF, ERBB2, EGFR, KRAS, and NRAS genes. In addition, we increased the down sampled coverage up to 5000 reads (the minimum reads randomly considered), allowing to improve the inter-run variability. Finally, we and others observed incorrect nomenclature calls [9]. To attribute the mutation position to the correct variant annotation, the “allele view” and the “allow complex” parameters were selected.

In order to validate our modifications of the bioinformatic pipeline, we analyzed the inter-run variability of two QC materials. We observed similar inter-run variabilities between the OFA and THS kits (Table 1). As expected, the variability was higher for low VAFs. However, the low coefficients of variation allowed the detection of mutations down to 1% VAF at each run. Furthermore, mutations from 3 to 5% expected VAF were systematically called (Table 1; Figure 2 and Figure S1). This long-term follow-up of VAF values highlighted that there were no loss of performance and no drift over time. This low variability was appropriate for clinical sample analysis. An inter-laboratory study involving six teams and analyzing the same QC materials obtained similar VAF for most mutations. However, we observed differences only for mutations with VAF < 5%. Five of the six laboratories did not report the EGFR p.Leu858Arg SNP with a VAF of 4% and none reported the EGFR p.Thr790Met and p.Glu746_Ala750del variations at VAF 1% and 2%, respectively [10]. This increased sensibility may be due to our optimization of the bioinformatic pipeline.

Using FFPE clinical samples, the observed inter-run variability was higher (Figure 3). These results, which were expected, were possibly due to lower DNA quality. Nevertheless, this larger variability remained sufficient to systematically report each expected mutation.

In our routine practice, we include a QC sample at each library preparation for quality purpose. Considering clinical results interpretation, to qualify a variant as both authentic and relevant, we take into consideration the number of reads at the corresponding genomic position, the tumor cellularity, and the clinical context.

In the case of patient follow-up or for investigation of therapeutic resistance mechanism, the communication to clinicians of variants with VAF below the threshold of 5% may have a significant impact on prognosis [13,14]. During the 21-month period of DNA analysis, we detected 353 mutations among tissue samples. The increased sensitivity of the bioinformatic analysis allowed the detection of 10 mutations with VAF below 3%. The mutation with the lowest VAF (KRAS p.Gly12Asp; 2%) was detected in a colon sample. When such low VAF mutation are detected, the biologist has to consider also detailed metrics and genomic alignment visualization to appreciate its theranostic relevance [13,14,15].

Considering gene amplification, the CNV value is interpreted according to the MAPD score. The analysis of inter-run variability using QC material revealed that both expected gene amplifications were detected and associated MAPD scores were below the threshold of 0.4, giving confidence to these results. The coefficients of variation were notably low for MET and MYCN amplification (5.2% and 3.7%, respectively), attesting the inter-run stability over time. In four clinical samples, ERBB2 gene amplifications were confirmed by immunocytochemistry (Table 3; Figure 5). This limited comparison suggests that, using OFA, the CNV represented more a confidence level of positivity than an absolute value [16]. In order to avoid false positive, we choose to report only CNV with a “5% confidence lower limit” above 6 [17]. Among the 88 amplifications called by the pipeline, we reported 79 (91%) results to the clinicians.

The scarcity of patients presenting RNA alteration imposed a validation protocol using two different QC materials in order to increase the diversity of fusion transcripts. The expected fusion transcripts were systematically detected throughout the 12-month period. Despite a high heterogeneity of determined inter-run variability CV, fusions reads counts were always above the manufacturer recommended thresholds (i.e., 20 for targeted fusions, 250 for non-targeted fusions, and 120 for exon skipping). Like others, we chose to determine the ratio of fusion reads counts to total mapped reads counts (Table 4) [10]. This ratio, similar to VAF, was useful to interpret clinical samples with low total RNA quantity. In our QC study, a fusion transcript could be reported with a percentage as low as 1% of total mapped reads. In contrast, in samples with a high amount of total RNA mapped reads, a ratio below 10% may indicate a false positive result.

Regarding HD784 QC material, we obtained similar fusion reads and fusion reads ratios to those reported by Bartlett et al.’s study [10]. In our clinical samples study, we reported seven fusions or exon skipping variants; among them, two were validated by immunochemistry (Figure 7).

Our results demonstrated that the sensitivity and the accuracy of OFA were suitable for molecular analysis of low quality and/or limited amounts of nucleic acids obtained from FFPE tumor samples in clinical routine practice. Slight modifications of the bioinformatic pipeline allowed to improve detection of low VAF variants, which may be useful to early detect genetic alterations involved in resistance mechanisms. This long-term study conducted in real-life conditions demonstrated that the performances of the OFA remained stable over time and ensured the reliability of results with theranostic impact.

## Figures and Tables

**Table 1:** Inter-run variability of VAF obtained with HD200 and HD300 QC materials on S5XL and MiSeq instruments.

		Oncomine Focus Assay				Tumor Hot Spot	
	Expected VAF (%)	n	Mean VAF (%) (Mean ± SD)	CV (%)	N	Mean VAF (%) (Mean ± SD)	CV (%)
*BRAF* p.Val600Glu	11	42	11.8 (11.1–12.5)	6.0	43	11.6 (9.8–13.4)	7.7
*EGFR* p.Glu746_Ala750del	2	42	1.5 (1.2–1.9)	22.4	43	1.9 (1.6–2.3)	19.2
*EGFR* p.Glu746_Ala750del	5	35	4.0 (3.4–4.5)	14.5	42	5.1 (4.5–5.7)	11.5
*EGFR* p.Gly719Ser	5	35	5.2 (4.5–6.0)	14.6	42	5.7 (5.2–6.2)	9.4
*EGFR* p.Thr790Met	1	42	1.1 (0.7–1.5)	37.7	43	1.2 (0.6–1.7)	46.5
*EGFR* p.Leu861Gln	5	35	5.5 (4.9–6.0)	10.3	42	4.7 (4.3–5.2)	10.0
*EGFR* p.Leu858Arg	3	42	3.4 (2.7–3.9)	13.3	43	3.4 (3.0–4.0)	14.1
*KRAS* p.Gly12Asp	6	42	6.2 (5.5–7.0)	12.3	43	6.5 (4.8–8.7)	12.9
*KRAS* p.Gly13Asp	15	42	14.2 (13.1–15.4)	8.1	43	15.2 (11.7–18.7)	11.5
*NRAS* p.Gln61Lys	13	42	12.9 (11.6–14.2)	9.8	43	11.8 (9.4–14.1)	10.0
*PIK3CA* p.Glu545Lys	9	42	7.5 (5.4–9.5)	11.9	43	8.3 (6.7–10.0)	10.1
*PIK3CA* p.His1047Arg	18	42	17.5 (16.4–18.4)	5.6	43	17.7 (15.3–20.1)	6.8

**Table 2:** Inter-run variation of gene amplification analysis using HD789 QC material in 20 consecutive runs.

Gene	Expected CNV	Measured CNV (Mean ± SD)	CV (%)	5% Limit Variability of OFA Confidence Interval (Mean ± SD)	95% Limit Variability of OFA Confidence Interval (Mean ± SD)
*MET*	4.5	5.8 ± 0.3	5.2%	4.5 ± 0.4	7.4 ± 0.4
*MYCN*	8.5	14.1 ± 1.0	3.7%	11.3 ± 1.0	17.6 ± 0.8

**Table 3:** *ERBB2* gene amplification evaluation by NGS and immunochemistry analysis.

Sample	MAPD	*ERBB2* CNV	CNV Confidence Interval (5–95%)	Immunochemistry Staining Intensity
Patient 1	0.323	28.0	22.2–35.2	Strong (+++)
Patient 2	0.360	9.5	7.5–12.0	Strong (+++)
Patient 3	0.282	5.4	4.2–6.8	Intermediate (++/+++)
Patient 4	0.334	12.0	8.3–16.5	Low (+)

**Table 4:** Inter-run variation for gene-fusion and exon skipping detection in QC samples.

**HD784 QC Material (*n* =** **29)**				
**Total Mapped Fusion Allele Reads: 95,753 (57,060–134,445); CV: 40.4%**				
	**Fusion Reads Counts**		**Fusion Reads (%)**	
	**Mean (Mean ± SD)**	**CV (%)**	**Mean (Mean ± SD)**	**CV (%)**
*CCDC6*(1)-*RET*(12)	4034 (1867–6212)	54.0	4.6 (3.5–5.7)	23.2
*SLC34A2*(4)-*ROS1*(32)	14,637 (7927–21,348)	45.8	17.8 (13.0–22.7)	27.1
*EML4*(13)-*ALK*(20)	4289 (1457–7121)	35.2	4.5 (2.9–6.1)	27.2
**Seracare QC Material (*n* =** **7)**				
**Total Mapped Fusion Allele Reads: 98,353 (73,474–** **123,233); CV: 23.3%**				
	**Fusion Reads Counts**		**Fusion Reads (%)**	
	**Mean (Mean ± SD)**	**CV (%)**	**Mean (Mean ± SD)**	**CV (%)**
*FGFR3*(17)-*BAIAP2L1*(2)	4436 (3849–5023)	13.2	4.6 (3.9–5.3)	15.1
*CCDC6*(1)-*RET*(12)	1589 (1194–1985)	24.9	1.6 (1.4–1.9)	17.5
*KIF5B*(24)-*RET*(11)	3639 (24,511–4828)	32.7	3.7 (2.9–4.5)	21.8
*NCOA4*(8)-*RE*T(12)	1706 (1458–1955)	14.6	1.8 (1.6–1.9)	9.5
*TMPRSS2*(1)-*ERG*(2)	3282 (1437–5126)	56.2	3.2 (2.2–4.2)	31.1
*PAX8*(9)-*PPARG*(3)	3909 (2476–5343)	36.7	3.9 (3.2–4.7)	18.1
*EML4*(13)*-ALK*(20)	2837 (2464–3211)	13.2	3 (2.3–3.7)	22.2
*SLC45A3*(1)*-BRAF*(8)	5436 (1681–9192)	69.1	5.2 (3.3–7.0)	36.0
*SLC34A2*(4) *-ROS1*(34)	3437 (2111–4761)	38.6	3.4 (2.9–4.0)	15.9
*CD74*(6)-*ROS1*(34)	7090 (5700–8479)	19.6	7.4 (5.8–8.9)	20.9
*LMNA*(2)-*NTRK1*(10)	1701 (531–2874)	68.8	1.6 (1.0–2.6)	38.4
*TFG*(5)-*NTRK1*(10)	1323 (923–1723)	30.2	1.3 (1.4–1.5)	14.9
*TPM3*(7)-*NTRK1*(9)	2822 (2127–3517)	24.6	2.9 (2.6–3.1)	7.9
*ETV6*(5)-*NTRK3*(15)	2352 (1908–2796)	18.9	2.4 (2.0–2.9)	17.4
*EGFR*(1)-*EGFR*(8) (vIII)	2605 (1390–3821)	46.6	2.6 (2.0–3.0)	18.3
*MET*(13)-*MET*(15)	2451 (2012–2889)	17.9	2.6 (1.9–3.3)	27.0

## Data Availability

Restrictions apply to the availability of these data. Data were obtained from Assistance Publique-Hôpitaux de Paris and are available from the authors with the permission of Assistance Publique-Hôpitaux de Paris.

## Supplementary Materials

The following supporting information can be downloaded at: https://www.mdpi.com/article/10.3390/diagnostics13050937/s1, Figure S1: Levey-Jennings plots of VAF obtained for five additional representative variants from HD200 and HD300 QC materials on S5XL instrument over a 21-months follow-up period; Figure S2: Immuno-cytochemistry analysis of ERBB2 protein in a non-tumoral bone tissue highlighting negative staining; Figure S3: Immuno-cytochemistry analysis of ROS1 protein in a non-tumoral lung tissue highlighting negative staining.

## Appendix A

Genomic position of the added clinically relevant hotspot mutations.

**Chromosome**	**Genomic position**		**Allele**	**Amplicon**
chr1	115252288	115252289	REF = C;OBS = T	OCP1_NRAS_1
chr1	115252288	115252289	REF = C;OBS = G	OCP1_NRAS_1
chr1	115252289	115252290	REF = T;OBS = A	OCP1_NRAS_1
chr1	115252289	115252290	REF = T;OBS = C	OCP1_NRAS_1
chr1	115252289	115252290	REF = T;OBS = G	OCP1_NRAS_1
chr1	115252290	115252291	REF = T;OBS = A	OCP1_NRAS_1
chr1	115252290	115252291	REF = T;OBS = C	OCP1_NRAS_1
chr1	115252290	115252291	REF = T;OBS = G	OCP1_NRAS_1
chr1	115256536	115256537	REF = T;OBS = A	CHP2_NRAS_2
chr1	115256536	115256537	REF = T;OBS = G	CHP2_NRAS_2
chr1	115256536	115256537	REF = T;OBS = C	CHP2_NRAS_2
chr1	115256537	115256538	REF = G;OBS = C	CHP2_NRAS_2
chr1	115256537	115256538	REF = G;OBS = T	CHP2_NRAS_2
chr1	115256537	115256538	REF = G;OBS = A	CHP2_NRAS_2
chr1	115256538	115256539	REF = T;OBS = C	CHP2_NRAS_2
chr1	115256538	115256539	REF = T;OBS = A	CHP2_NRAS_2
chr1	115256538	115256539	REF = T;OBS = G	CHP2_NRAS_2
chr7	140453137	140453138	REF = T;OBS = A	CHP2_BRAF_2
chr7	140453137	140453138	REF = T;OBS = C	CHP2_BRAF_2
chr7	140453137	140453138	REF = T;OBS = G	CHP2_BRAF_2
chr7	140453138	140453139	REF = G;OBS = A	CHP2_BRAF_2
chr7	140453138	140453139	REF = G;OBS = C	CHP2_BRAF_2
chr7	140453138	140453139	REF = G;OBS = T	CHP2_BRAF_2
chr7	140453139	140453140	REF = T;OBS = A	CHP2_BRAF_2
chr7	140453139	140453140	REF = T;OBS = C	CHP2_BRAF_2
chr7	140453139	140453140	REF = T;OBS = G	CHP2_BRAF_2
chr7	140453140	140453141	REF = A;OBS = G	CHP2_BRAF_2
chr7	140453140	140453141	REF = A;OBS = T	CHP2_BRAF_2
chr7	140453140	140453141	REF = A;OBS = C	CHP2_BRAF_2
chr7	140453140	140453141	REF = A;OBS = C	CHP2_BRAF_2
chr7	140481415	140481416	REF = T;OBS = A	OCP1_BRAF_1
chr7	140481415	140481416	REF = T;OBS = G	OCP1_BRAF_1
chr7	140481415	140481416	REF = T;OBS = C	OCP1_BRAF_1
chr7	140481416	140481417	REF = C;OBS = T	OCP1_BRAF_1
chr7	140481416	140481417	REF = C;OBS = A	OCP1_BRAF_1
chr7	140481416	140481417	REF = C;OBS = G	OCP1_BRAF_1
chr7	140481417	140481418	REF = C;OBS = G	OCP1_BRAF_1
chr7	140481417	140481418	REF = C;OBS = A	OCP1_BRAF_1
chr7	140481417	140481418	REF = C;OBS = T	OCP1_BRAF_1
chr12	25378556	25378557	REF = C;OBS = A	CHP2_KRAS_3
chr12	25378556	25378557	REF = C;OBS = T	CHP2_KRAS_3
chr12	25378556	25378557	REF = C;OBS = G	CHP2_KRAS_3
chr12	25378557	25378558	REF = T;OBS = A	CHP2_KRAS_3
chr12	25378557	25378558	REF = T;OBS = C	CHP2_KRAS_3
chr12	25378557	25378558	REF = T;OBS = G	CHP2_KRAS_3
chr12	25378558	25378559	REF = T;OBS = A	CHP2_KRAS_3
chr12	25378558	25378559	REF = T;OBS = C	CHP2_KRAS_3

## Appendix B

Amplicon ID list:
1.OCP1_AR_8, 2.OCP1_DCUN1D1_5, 3.OCP1_AR_10, 4.OCP1_AR_2, 5.OCP1_DCUN1D1_9, 6.OCP1_MYCN_2, 7.Oncomine_Focus_PIK3CA_1, 8.OCP1_KRAS_10, 9.OCP1_MYCN_14, 10.OCP1_NF1_49, 11.OCP1_FGFR3_12, 12.OCP1_AR_16, 13.Oncomine_Focus_BRAF_5, 14.OCP1_KRAS_1, 15.OCP1_FGFR2_12, 16.OCP1_FGFR2_16, 17.OCP1_EGFR_13, 18.OCP1_FGFR3_3, 19.OCP1_PDGFRA_4, 20.OCP1_MED12_3, 21.OCP1_FGFR3_1, 22.OCP1_AR_6, 23.OCP1_FGFR2_8, 24.ON_DDR2_2, 25.OCP1_CDK4_2, 26.OCP1_KIT_10, 27.OCP1_EGFR_16, 28.Oncomine_Focus_BRAF_7, 29.Oncomine_Focus_ALK_1, 30.OCP1_DCUN1D1_3, 31.OCP1_AR_3, 32.OCP1_KIT_1, 33.OCP1_BRCA1_91, 34.OCP1_NF1_1, 35.OCP1_AR_12, 36.OCP1_FGFR4_5, 37.OCP1_BIRC2_11, 38.OCP1_BRCA1_84, 39.OCP1_MYC_5, 40.OCP1_MED12_1, 41.OCP1_MYCN_12, 42.OCP1_KIT_7, 43.OCP1_CDK6_2, 44.OCP1_CCND1_1, 45.OCP1_AR_11, 46.CHP2_CTNNB1_1, 47.OCP1_MYCN_19, 48.OCP1_AR_14, 49.OCP1_MYCN_10, 50.CHP2_FGFR3_3, 51.OCP1_NF1_89, 52.OCP1_PDGFRA_18, 53.CHP2_SMO_3, 54.OCP1_KRAS_4, 55.OCP1_MTOR_2, 56.OCP1_BRCA1_6, 57.OCP1_PDGFRA_10, 58.CHP2_NRAS_1, 59.OCP1_CDK6_13, 60.OCP1_MYC_3, 61.OCP1_CCND1_10, 62.OCP1_MET_13, 63.OCP1_GNAQ_1, 64.OCP1_MYC_11, 65.Oncomine_Focus_PIK3CA_2, 66.OCP1_CDK4_8, 67.OCP1_FGFR4_16, 68.Oncomine_Focus_ALK_6, 69.CHP2_PIK3CA_10, 70.OCP1_ESR1_3, 71.OCP1_AR_15, 72.OCP1_MYCN_1, 73.CHP2_FGFR2_1, 74.CHP2_FGFR3_4, 75.OCP1_ERBB2_22, 76.OCP1_MET_16, 77.ON_EGFR_2A, 78.OCP1_FGFR2_18, 79.OCP1_MET_1, 80.Oncomine_Focus_ALK_5, 81.OCP1_CCND1_6, 82.OCP1_FGFR4_17, 83.OCP1_MYCN_9, 84.OCP1_CCND1_4, 85.OCP1_CCND1_18, 86.OCP1_MTOR_4, 87.OCP1_CCND1_7, 88.OCP1_APC_32, 89.OCP1_MYC_6, 90.CCP_EGFR_6, 91.OCP1_FGFR1_2, 92.CHP2_FGFR3_2, 93.OCP1_ERBB2_14, 94.OCP1_FGFR4_13, 95.OCP1_JAK3_2, 96.OCP1_CDK4_14, 97.OCP1_PDGFRA_7, 98.OCP1_DCUN1D1_10, 99.OCP1_BIRC2_12, 100.Oncomine_Focus_MTOR_3, 101.OCP1_MET_19, 102.OCP1_PIK3CA_18, 103.Oncomine_Focus_ALK_7, 104.OCP1_APC_40, 105.CHP2_MET_5, 106.Oncomine_Focus_AR_1, 107.OCP1_KRAS_8, 108.OCP1_FGFR1_17, 109.OCP1_FGFR4_2, 110.OCP1_FGFR4_18, 111.OCP1_ALK_1, 112.OCP1_RAF1_1, 113.CHP2_GNA11_1, 114.OCP1_KRAS_5, 115.OCP1_CDK6_14, 116.OCP1_MYCN_6, 117.OCP1_FGFR1_10, 118.Oncomine_Focus_HRAS_1, 119.CHP2_KIT_5, 120.OCP1_MYC_4, 121.OCP1_BIRC2_8, 122.OCP1_FGFR3_8, 123.CHP2_SMO_4, 124.OCP1_FGFR1_18, 125.OCP1_FGFR2_13, 126.OCP1_CDK6_5, 127.OCP1_NRAS_1, 128.OCP1_BIRC2_14, 129.CHP2_RET_2, 130.ON_MAP2K1_1, 131.CHP2_IDH2_1, 132.OCP1_MET_5, 133.CHP2_PDGFRA_2, 134.CHP2_RET_3, 135.OCP1_JAK1_1, 136.OCP1_ERBB2_23, 137.CHP2_PIK3CA_6, 138.OCP1_KRAS_7, 139.Oncomine_Focus_ALK_4, 140.OCP1_CCND1_17, 141.OCP1_FGFR1_8, 142.CHP2_KRAS_3, 143.CHP2_GNAQ_1, 144.Oncomine_Focus_PIK3CA_3, 145.OCP1_FGFR1_13, 146.CHP2_BRAF_2, 147.CHP2_NRAS_2, 148.Oncomine_Focus_GNA11_1, 149.OCP1_CDK6_15, 150.CHP2_FGFR2_4, 151.OCP1_CCND1_8, 152.Oncomine_Focus_BRAF_4, 153.OCP1_MYC_1, 154.OCP1_ERBB3_1, 155.OCP1_NF1_130, 156.OCP1_MYC_2, 157.Oncomine_Focus_MTOR_2, 158.Oncomine_Focus_MET_1, 159.CHP2_FGFR1_2, 160.OCP1_ALK_2, 161.CHP2_RET_4, 162.OCP1_CDK4_6, 163.OCP1_BRCA1_88, 164.OCP1_ERBB2_12, 165.Oncomine_Focus_SMO_1, 166.Oncomine_Focus_MTOR_4, 167.OCP1_MTOR_3, 168.OCP1_APC_1, 169.CHP2_KIT_2, 170.OCP1_MAP2K1_2, 171.OCP1_CDK4_7, 172.OCP1_FGFR3_13, 173.CHP2_AKT1_1, 174.CHP2_HRAS_2, 175.Oncomine_Focus_SMO_2, 176.CHP2_RET_1, 177.Oncomine_Focus_ROS1_1, 178.OCP1_KRAS_6, 179.OCP1_KIT_13, 180.Oncomine_Focus_FGFR2_1, 181.Oncomine_Focus_FGFR2_2, 182.OCP1_CDK6_4, 183.OCP1_BRAF_1, 184.OCP1_CCND1_9, 185.CHP2_FGFR3_1, 186.OCP1_APC_62, 187.OCP1_MYCN_13, 188.OCP1_FGFR4_11, 189.CHP2_ERBB2_2, 190.OCP1_BRCA1_11, 191.OCP1_CDK4_4, 192.CHP2_MET_3, 193.OCP1_CDK6_6, 194.Oncomine_Focus_KIT_1, 195.OCP1_BIRC2_7, 196.OCP1_KIT_20, 197.Oncomine_Focus_RAF1_1, 198.OCP1_CDK4_13, 199.OCP1_MYC_7, 200.CHP2_EGFR_3, 201.OCP1_JAK3_1, 202.CHP2_EGFR_4, 203.OCP1_FGFR4_10, 204.Oncomine_Focus_MTOR_1, 205.CHP2_RET_5, 206.Oncomine_Focus_ERBB2_2, 207.OCP1_CCND1_2, 208.Oncomine_Focus_BRAF_8, 209.OCP1_DCUN1D1_12, 210.OCP1_FGFR4_8, 211.Oncomine_Focus_ROS1_2, 212.OCP1_CDK4_12, 213.Oncomine_Focus_BRAF_6, 214.CHP2_PIK3CA_9, 215.CHP2_MET_6, 216.OCP1_MAP2K2_1, 217.OCP1_MYC_10, 218.OCP1_FGFR3_5, 219.Oncomine_Focus_BRAF_3, 220.CHP2_EGFR_2, 221.OCP1_ERBB2_17, 222.Oncomine_Focus_BRAF_1, 223.OCP1_FGFR1_7, 224.ON_ALK_0, 225.CHP2_KIT_3, 226.CHP2_FGFR2_3, 227.Oncomine_Focus_ERBB3_1, 228.CHP2_PDGFRA_1, 229.CHP2_JAK2_1, 230.OCP1_CDK4_3, 231.Oncomine_Focus_ALK_2, 232. Oncomine_Focus_JAK1_1, 233. OCP1_JAK1_2, 234. OCP1_FGFR1_16, 235. Oncomine_Focus_ERBB2_1, 236. OCP1_PDGFRA_6, 237. OCP1_MYC_8, 238. OCP1_MET_8, 239. OCP1_FGFR4_4, 240. OCP1_PIK3CA_19, 241. CHP2_EGFR_1, 242. OCP1_ERBB3_2, 243. OCP1_CDK6_12, 244. CHP2_PIK3CA_3, 245. OCP1_PDGFRA_12, 246. OCP1_FGFR1_1, 247. CHP2_KRAS_1, 248. OCP1_CDK6_7, 249. Oncomine_Focus_JAK3_1, 250. Oncomine_Focus_ALK_3, 251. OCP1_PDGFRA_2, 252. OCP1_EGFR_23, 253. Oncomine_Focus_BRAF_2, 254. CHP2_IDH1_1, 255. OCP1_MAP2K1_1, 256. OCP1_MYCN_8, 257. Oncomine_Focus_ERBB4_1, 258. OCP1_PIK3CA_20, 259. CHP2_ERBB2_3, 260. OCP1_CDK6_11, 261. Oncomine_Focus_ERBB3_2, 262. OCP1_APC_21, 263. Oncomine_Focus_ERBB3_3, 264. CHP2_PDGFRA_4, 265. OCP1_CDK4_10, 266. CHP2_KRAS_2, 267. CHP2_ERBB2_1, 268. CHP2_EGFR_5, 269. CHP2_KIT_4.
