# Supplementary material for: Performance Characteristics of Oncomine Focus Assay for Theranostic Analysis of Solid Tumors, A (21-Months) Real-Life Study
Source: Diagnostics (Basel). 2023 Mar 1;13(5):937. doi: 10.3390/diagnostics13050937 (PMC10001101; doi:10.3390/diagnostics13050937)
Supplement: Supplementary file 1 [file diagnostics-13-00937-s001.zip › diagnostics-2149699-supplementary.pdf]

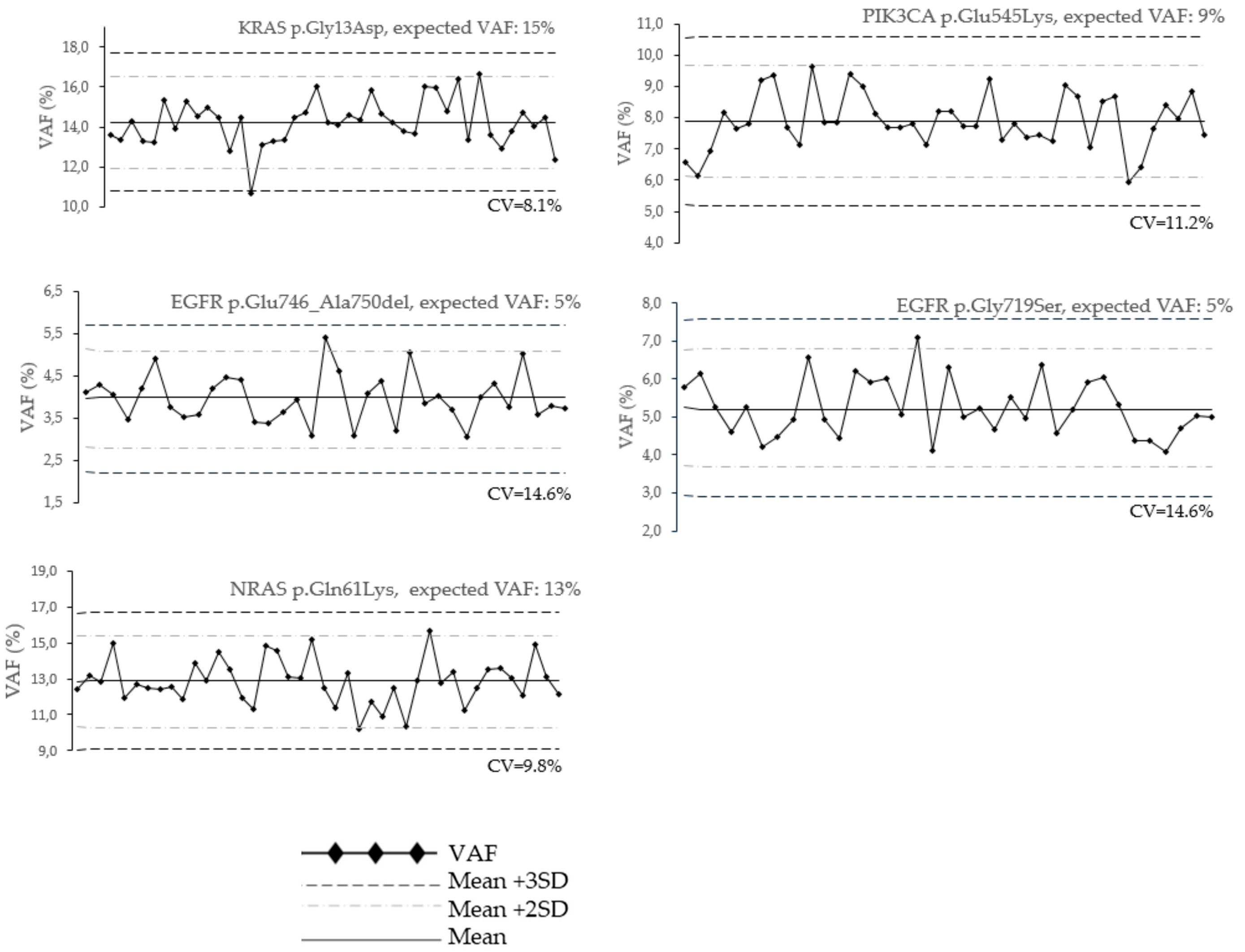

Figure S1. Levey-Jennings plots of VAF obtained for five additional representative variants from HD200 and HD300 QC materials on S5XL instrument over a 21-months follow-up period.

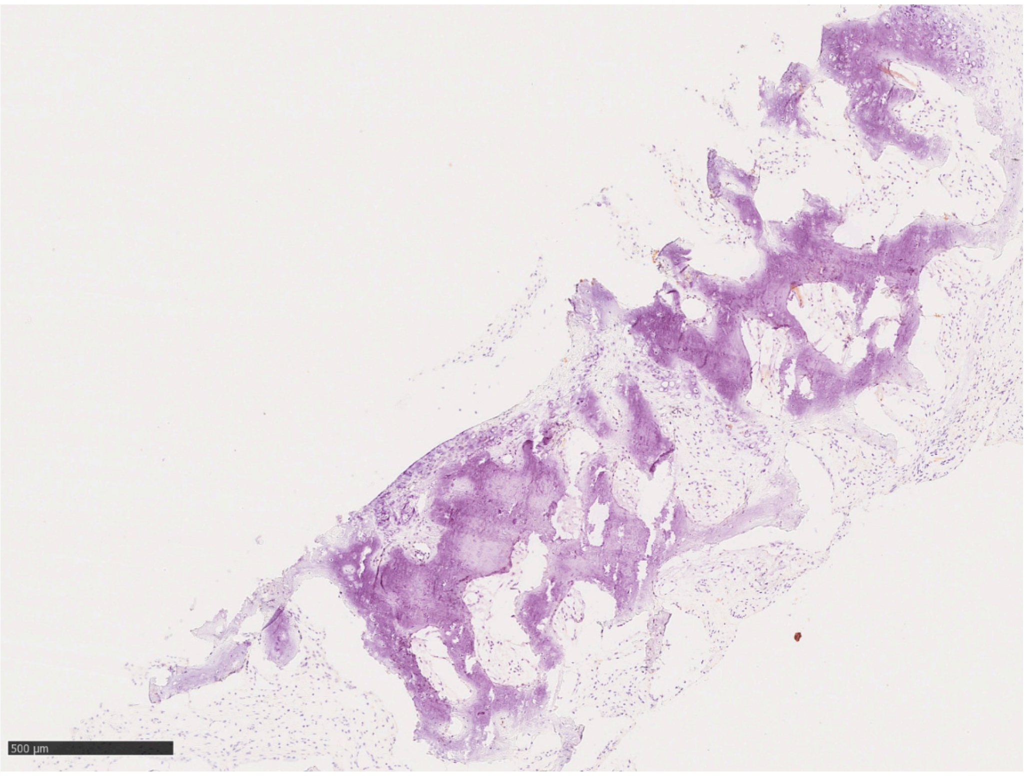

Figure S2. Immuno-cytochemistry analysis of ERBB2 protein in a non-tumoral bone tissue highlighting negative staining.

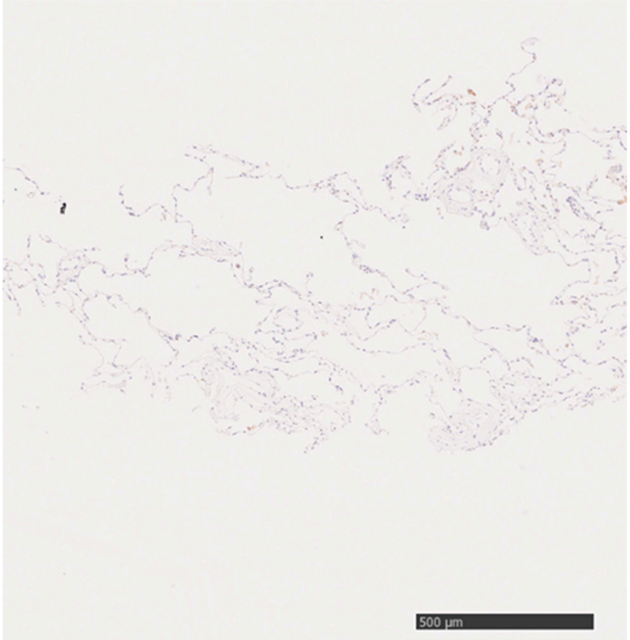

Figure S3. Immuno-cytochemistry analysis of ROS1 protein in a non-tumoral lung tissue highlighting negative staining.
